# Fetal and neonatal programming of postnatal growth and feed efficiency in swine

**DOI:** 10.1186/s40104-017-0173-5

**Published:** 2017-05-05

**Authors:** Yun Ji, Zhenlong Wu, Zhaolai Dai, Xiaolong Wang, Ju Li, Binggen Wang, Guoyao Wu

**Affiliations:** 1 State Key Laboratory of Animal Nutrition, China Agricultural University, Beijing, 100193 China; 2 Henan Yinfa Animal Husbandry Co., Ltd., Xinzheng, Henan 451100 China; 3 Department of Animal Science and Center for Animal Genomics, Texas A&M University, Room 212, College Station, TX 77843 USA

**Keywords:** Epigenetics, Fetal programming, Gene expression, Neonatal programming, Nutrition

## Abstract

Maternal undernutrition or overnutrition during pregnancy alters organ structure, impairs prenatal and neonatal growth and development, and reduces feed efficiency for lean tissue gains in pigs. These adverse effects may be carried over to the next generation or beyond. This phenomenon of the transgenerational impacts is known as fetal programming, which is mediated by stable and heritable alterations of gene expression through covalent modifications of DNA and histones without changes in DNA sequences (namely, epigenetics). The mechanisms responsible for the epigenetic regulation of protein expression and functions include chromatin remodeling; DNA methylation (occurring at the 5´-position of cytosine residues within CpG dinucleotides); and histone modifications (acetylation, methylation, phosphorylation, and ubiquitination). Like maternal malnutrition, undernutrition during the neonatal period also reduces growth performance and feed efficiency (weight gain:feed intake; also known as weight-gain efficiency) in postweaning pigs by 5–10%, thereby increasing the days necessary to reach the market body-weight. Supplementing functional amino acids (e.g., arginine and glutamine) and vitamins (e.g., folate) play a key role in activating the mammalian target of rapamycin signaling and regulating the provision of methyl donors for DNA and protein methylation. Therefore, these nutrients are beneficial for the dietary treatment of metabolic disorders in offspring with intrauterine growth restriction or neonatal malnutrition. The mechanism-based strategies hold great promise for the improvement of the efficiency of pork production and the sustainability of the global swine industry.

## Background

The gestational (114 d) and neonatal (21 d after birth) periods are two critical phases in swine production [1]. Among livestock mammals, pigs exhibit the highest rates of embryonic mortality, intrauterine growth restriction (IUGR), and neonatal deaths [2–4]. These problems are exacerbated by a variety of factors encountered in the different phases of swine production, including extreme ranges of environmental temperatures, feed hygiene and safety, suboptimal nutrition, and disease [5]. When adverse conditions occur during gestation or nursing, the negative impacts on the offspring can last for their entire life cycle and can be carried over to the next generation or beyond [6]. This concept is called fetal or neonatal programming [7], which involves the covalent modifications of nucleotide bases in DNAs without changes in their sequences [8–10]. Thus, the global swine industry must overcome enormous challenges to achieve a high efficiency of pork production and high economic returns. One approach is to optimize the nutrition of the mothers and neonates [6, 11]. The main objective of this article is to highlight and integrate the complex aspects of swine biological characteristics, IUGR, as well as the fetal and neonatal programming of postnatal growth and feed efficiency (weight gain:feed intake) in pigs.

## Unique biological characteristics of swine relevant to nutrition

**Fig. 1:**
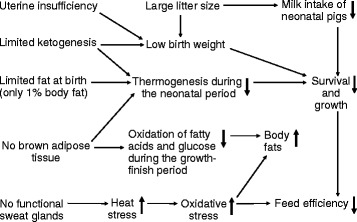
Unique biological characteristics of swine that differ from livestock ruminant species. Pigs possess no brown adipose tissue (BAT), limited ketogenesis, and a limited amount of fetal fats, which result in a low rate of thermogenesis during the neonatal period. Both uterine insufficiency and large litter size due to genetic selection contribute to intrauterine growth restriction. With a large number of piglets and no increase in the lactation performance of sows, milk consumption by them is inadequate for their maximum survival and growth. Failure to maintain body temperature or receive adequate nutritional support results in the high rates of morbidity and mortality in neonatal pigs. On the other hand, pigs are susceptible to heat stress due to their lack of functional sweat glands, and, therefore, exhibit the enhanced production of oxygen free radicals in response to high ambient temperatures. Their oxidative stress and lack of BAT promote fat deposition in the body. Both oxidative stress and mortality decrease feed efficiency in pigs. The signs “↓” and “↑” denote decrease and increase, respectively

Embryos, fetuses, and neonates of all species are very sensitive to the detrimental effects of high ammonia concentrations in their plasma [10]. Adequate knowledge of the biology of swine is essential to understanding their nutrition and developing effective methods for improving their growth and survival. Pigs have some distinct biological characteristics that are very different from other livestock species (Fig. 1). These features should be considered in dietary formulations and managements of both normal-birth weight and IUGR piglets.

First, the pig has no brown adipose tissue (BAT) in its life cycle to oxidize long-chain fatty acids and glucose [12]. Thus, there is no production of non-shivering heat in neonatal pigs, therefore contributing to the development of hypothermia and high rates of mortality in them, particularly in IUGR piglets which have a higher risk for metabolic disorders (including hypoglycemia) than normal-birth weight piglets [1]. On the other hand, the dam gains excessive amounts of maternal subcutaneous white adipose tissue (WAT) during pregnancy when fed either ad libitum or >50% of ad libitum feed intake [13]. While this metabolic arrangement may be beneficial for conserving maternal fat reserves in the face of limited food intake during species evolution, the adverse impacts of such a maternal adaptation mechanism include a high rate of embryonic/fetal mortality during early gestation [14]. In addition, sows with excess WAT exhibit impaired lactation performance post-partum [1]. Therefore, in the modern swine industry, gestating gilts are fed only a 2 to 2.2 kg diet (containing 12% CP) daily, which is only ~50% of their ad libitum feed intake, to minimize maternal WAT accretion. Clearly, this is a case of underfeeding in nutritional terms. The production-imposed reduction in the feed intake of pregnant gilts results in the inadequate provision of amino acids (AAs) in the gestation diets, thereby impairing fetal-pig growth and development [15]. Three of four nutritionally important AAs are L-arginine, L-glutamine, glycine, and L-proline for gestating gilts [1]. Thus, the unique characteristics of fat metabolism in swine limit their dietary intakes of energy and AAs during the entire period of pregnancy, therefore increasing the risk for IUGR and negatively impacting the vigor, survival, growth and feed efficiency of postnatal pigs.

Second, pigs have limited ketogenesis (the production of acetoacetate and β-hydroxybutyrate from fatty acids) at any developmental stage due to their low expression of hepatic 3-hydroxy-3-methylglutaryl-CoA (HMG-CoA) synthase [16, 17]. Thus, pigs have very low concentrations of ketone bodies (<0.2 mmol/L) in plasma under either fed or food-deprived conditions. Although acetoacetate and β-hydroxybutyrate are actively utilized for ATP production by extra-hepatic tissues, such as the brain, skeletal muscle, heart, kidneys, and small intestine in mammals (including pigs) [18], the endogenously-generated ketone bodies only contribute a negligible amount of energy for swine. Consequently, in response to fasting or infections, when the concentration of glucose in plasma is substantially reduced, this sugar can only supply ~ 40% of the energy normally required by the brain [19]. This leads to impairments in neurological function and animal behavior, as well as increases in the rates of morbidity and mortality in pigs, particularly during the neonatal period [18]. When piglets die, their feed efficiency is zero.

Third, fetal pigs have a limited synthesis of fatty acids and triacylglycerols (TAG) [20]. Thus, at birth, the piglet has only 1% lipids in its body [21], which is in contrast to many other mammalian newborns (e.g., humans and cattle), which possess 10% or more fats [22]. The exceedingly small amount of endogenous lipids limits ATP production from these nutrients in neonatal pigs, leading to the use of glucose and AAs as the major metabolic fuels. This, along with the near absence of ketogenesis, readily results in hypoglycemia and the reduced availability of AAs for protein synthesis, particularly when milk intake is insufficient [23]. Additionally, because subcutaneous fats can be used for insulation, the limited amount of fats in newborn pigs further increases the risk for hypothermia and, therefore, mortality in them, especially in a cold environment [14]. Deaths of preweaning piglets will result in no feed value for sows. The ability to synthesize TAG (occurring primarily in WAT) rapidly develops in well-fed piglets after birth, which results in a body-lipid content of 14% at 2 weeks of age [24]. Compared with pigs with a normal birth weight, surviving IUGR pigs have a greater capacity for TAG deposition in the body during the growing-finishing period and, therefore, exhibit a lower feed efficiency and lower quality pork [14].

Fourth, pigs possess no functional sweat glands and, therefore, must use the inadequate mechanism of panting to dissipate heat [25]. Thus, growing-finishing, gestating or lactating swine are highly susceptible to heat stress, as well as the associated oxidative stress, resulting in an excessive production of reactive oxygen species [26, 27]. Under experimental conditions, increasing environmental temperatures from 23 °C to 33 °C markedly reduces feed intake, growth performance, and the efficiency of nutrient utilization for weight gains in swine [28–30], and, at the same time, impairs immune responses [31] and muscle protein synthesis [32]. Interestingly, the pig’s breed does not influence physiological responses to thermal stress [33, 34]. Thus, pigs raised in naturally warm areas exhibit poor production performance, including low daily weight gain [35], low protein deposition in muscle [36], poor lactation, and low litter size [37]. To date, a climate change towards global warming is expected to negatively impact swine production. Mader et al. [37] estimated that, with CO_2_ doubling and tripling, the time necessary for raising pigs from 50 to 110 kg in warm seasons would be increased by up to 30% and 74%, respectively, in the southernmost regions of the U.S. The lack of sweat glands in swine presents a challenge to design nutritional means for improving feed efficiency in growing pigs, particularly in IUGR pigs raised at high ambient temperatures.

## Intrauterine Growth Restriction (IUGR) in swine

IUGR is defined as impaired growth and development of the mammalian embryo/fetus or its organs during pregnancy [14]. In animal studies, IUGR is identified as fetal or birth weight less than two standard deviations of the mean body weight for gestational age. For crossbred sows (Yorkshire × Landrace dams and Duroc × Hampshire boars), the mean birth weight is 1.4 kg, and a piglet with a birth weight of less than 1.1 kg can be considered to have IUGR. Despite advances in nutrition and management techniques, IUGR remains a significant problem in swine production [3, 4]. Before d 35 of gestation, porcine embryos are uniformly distributed within each uterine horn. After this time in gestation, uterine capacity becomes a limiting factor for fetal growth even though the fetuses are distributed relatively uniformly [38]. Additionally, the proportion of low birth weight pigs at farrowing has increased in recent years due to the successful genetic selection for increased litter size, which results in increased uterine crowding and the associated decrease in placental weight per fetus [3, 39]. Approximately 24% of newborn piglets from gilts fed a 12%-CP diet have a birth weight of < 1.1 kg [5]. In some litters, most or nearly all of the piglets have reduced birth weights (<1.1 kg), particularly when a part or majority of the pregnancy period is subjected to environmental stress (e.g., hot or cold temperatures or infections) [40]. At birth, runt piglets may weigh only one-half or even one-third as much as their largest littermates, and key organs involved in nutrient digestion and utilization in runt pigs are disproportionately smaller than those of the larger littermates [41]. Thus, a major goal of feeding is to enhance piglet birth weight and reduce its variation within litters [39].

Most IUGR piglets die before weaning, and IUGR piglets (<1.10-kg birth weights) account for 76% of preweaning deaths in pigs [5]. IUGR has permanent negative impacts on organ structure, neonatal adjustment, preweaning survival, postnatal growth, feed efficiency, lifetime health, skeletal-muscle composition, excessive accumulation of WAT, meat quality, reproductive performance, and the onset of adult diseases [14, 42]. Altered organ mass and structure, such as reduced numbers of pancreatic islets, reduced numbers of kidney glomeruli, or reduced numbers of muscle fibers, is one of the consequences of fetal programming and is equally important to functional consequences. At present, farms will cull all IUGR piglets, and there is no nutritional support to increase their growth or survival during the suckling and postweaning periods [39]. Thus, although IUGR may be considered to be a natural mechanism to protect the dam in case of maternal undernutrition, it has adverse effects on the survival and growth performance of the progeny and the efficiency of pig production [43].

## Factors contributing to IUGR in swine

**Fig. 2:**
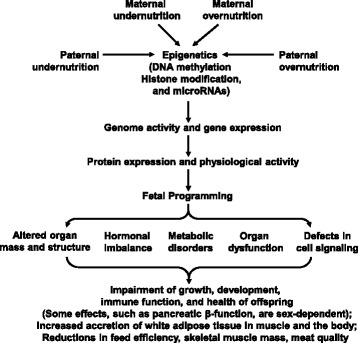
Genetic and environmental factors affecting fetal growth and development in swine. Either undernutrition or overnutrition of both the mother and father will affect the expression of the fetal genome, which may have lifelong consequences on the offspring. Thus, fetal malnutrition results in developmental adaptations that permanently change the structure, physiology and metabolism of the offspring. This predisposes the affected individuals to reductions in growth performance, skeletal-muscle mass, feed efficiency, as well as metabolic, endocrine, and cardiovascular disorders

Genetic (maternal and paternal), epigenetic, and environmental factors (including nutrition, ambient temperature, social stress and disease), as well as maternal maturity are all factors which affect fetal growth [9]. These factors also have an impact on the size and functional capacity of the placenta, placental vascular growth, uteroplacental blood flows, the transfer of nutrients from mother to fetus, the endocrine milieu, as well as embryonic and fetal development of myocytes, adipocytes and other cell types (Fig. 2). In a given breed, nutrition (low or high intake of dietary CP), disease, and environmental temperature are the three major determinants of IUGR [2]. Of particular note, porcine fetal growth can be negatively influenced by a severe maternal protein-energy imbalance during pregnancy [44]. Malnutrition during early gestation has a greater detrimental effect on fetal organ development than during mid- or late gestation [10].

## Maternal undernutrition and IUGR

### Undernutrition before breeding or during the periconceptual period

Much evidence shows that maternal malnutrition immediately before breeding or during the periconceptual period negatively affects oocyte quality, embryonic development and survival, and in fact ‘programs’ the timing of delivery of the newborn [45]. Insufficient feed intake remains a significant problem for lactating sows before breeding, because the mobilization of nutrient reserves for milk production results in a severe catabolic state and a prolonged interval from farrowing to estrus [39, 43]. Feed consumption of sows during lactation can be as low as 70% of the NRC requirements [46]. Inadequate nutrition increases the losses of body weight (BW) and backfat in lactating sows and also prolongs weaning-to-estrus intervals [46]. When sows enter pregnancy, the suboptimal nutritional status (namely premating maternal undernutrition), coupled with restricted feed intake, impairs the growth and development of early embryos and fetuses [2, 13, 15]. For example, in primiparous sows, restricting feed intake by 50% during lactation (a reduction from 5.0 to 2.5 kg/d between d 14 and d 21 of lactation) before mating reduces the weight of both male and female fetuses, as well as the survival of female embryos at d 30 of gestation [47]. Furthermore, reducing the intake of a complete ration by 50% for 2 estrous cycles before mating decreases fetal weight at d 30 of pregnancy in gilts [48]. Likewise, maternal undernutrition during the periconceptual period or the entire gestation reduces the fetal growth of pigs [49]. When the dietary provision of water, vitamins and minerals is sufficient, it is energy and protein/AA intake that are the major nutritional factors affecting fetal growth.

### Undernutrition during late gestation

Complete fasting of pregnant gilts for 10 d between d 100 and d 114 of gestation does not influence the body fat of fetuses due to their naturally limited ability for prenatal fat synthesis [50]. Of interest, fasting during the last 20 d of gestation increases the fat content of fetal adipose tissue [20], possibly due to the increased transfer of fatty acids from the mother to her fetuses. Decreasing feed intake by 28% after d 80 of gestation (2.5 vs. 1.8 kg feed/d) reduces fetal growth in gilts [51]. Because most fetal growth occurs in the last 3 weeks of pregnancy, undernutrition during late gestation has a greater negative effect on the birth weight of pigs than during early- or mid- gestation [14].

### Insulin resistance during late gestation

Maternal insulin resistance gradually develops in most mammals (including sows) late in their pregnancy [52]. This is likely due to the inability of the liver and skeletal muscle to oxidize the fatty acids released from WAT in response to a negative energy balance. An increase in plasma and tissue levels of free fatty acids is a major factor contributing to the occurrence of insulin resistance by reducing the synthesis of nitric oxide (NO) from arginine [18]. The low glucose tolerance of pregnant sows is associated with a high postnatal mortality of piglets and reduced efficiency of pork production [52]. Although insulin resistance in the dam may have the potential to increase the availability of glucose and AAs for the fetus, the transfer of nutrients from mother to fetus due to reduced placental blood flow is impaired under this metabolic condition. Because insulin stimulates muscle protein synthesis and inhibits muscle protein degradation, insulin resistance increases the net rate of whole-body proteolysis and thus plasma levels of methylarginines (inhibitors of endothelial NO synthesis). As NO is the major regulator of uteroplacental blood flow, severe insulin resistance decreases the placental delivery of nutrients and oxygen to the fetus during late gestation [5].

### Undernutrition during the entire gestation

The birth weight of piglets decreases in response to restricted feed intake during the entire gestation period (e.g., 0.9 vs. 1.9 kg/d) [53]. In addition, compared with adequate protein feeding, birth weights as well as brain and liver weights are reduced and bone development is impaired, in the progeny of gilts fed a protein-deficient diet throughout gestation [44, 49]. Likewise, severe maternal protein undernutrition during the entire gestation impairs the development of fetal skeletal-muscle fibers in pigs [54]. Besides the impairment of fetal growth (including bone growth), maternal undernutrition during the entire gestation will greatly reduce the longevity of the sows [55]. This has important implications for the sustainability of the swine industry for the following reasons. First, the production efficiency of a sow is determined by the number of piglets born alive and weaned per litter within a year during her breeding lifetime [39]. Second, litter size and piglet birth weights increase until the fourth or fifth parities, and the number of pigs weaned per sow within a year increases until the sixth and seventh parities [3, 39]. Third, healthy replacement gilts with the satisfactory nutrition status will most likely reach their fourth parity, when they are most productive for the swine operation [55].

In contrast to ruminants and horses, the pig generally has a remarkably high capacity to mobilize maternal energy reserves to support placental and fetal development during prolonged inanition in the presence of adequate progesterone and estrogen [56]. Thus, a modest reduction in the dietary intake of energy alone is not sufficient to cause IUGR in pigs. For example, in low-prolific gilts fed adequate amounts of protein, vitamins and minerals, restriction of dietary energy intake (50% of controls; namely 1.82 vs. 0.91 kg diet/d) does not affect the birth weight of piglets [44]. This likely results from the mobilization of TAG from the maternal WAT to spare AAs and glucose for fetal utilization. However, a more severe reduction in energy intake by gilts during the entire gestation from 8.0 to 2.2 Mcal of DE/d reduces birth weight, the number of skeletal-muscle fibers, muscle weight, liver weight, liver glycogen content, and serum protein concentrations in newborn piglets [57].

## Maternal overnutrition and IUGR

### Overnutrition before breeding or during the periconceptual period

Increasing energy intake increases the rate of ovulation in farm animals, including pigs [43]. Thus, the practice of increasing feed intake for a short period of time (termed flushing) around the time of conception was employed previously by producers in an attempt to increase the number of embryos/fetuses [55]. The practice of flushing is no longer recommended for swine producers because of concern over embryonic death [3, 4]. Overnutrition of gilts and sows can result from an increased intake of energy, protein, or both, which can occur before breeding or during pregnancy. Much evidence shows that high energy intake immediately before pregnancy and/or during early gestation increases embryonic mortality and reduces embryonic growth in swine [43, 55]. Similar results have been reported for gilts or sows consuming high amounts of dietary protein or a combination of high energy plus high protein either immediately before breeding or during early gestation [43, 48]. Disappointingly, to date, gestating gilts and sows are still frequently fed, on many commercial farms, relatively high-protein diets (e.g., 14% to 18% CP) throughout pregnancy. A diet containing 14% CP for feeding pregnant swine during the entire period of gestation is still currently used on some farms [58] and is also recommended by some extension agents [59], but such a gestation diet is not optimum for porcine embryonic survival (Table 1). This is because the adverse effects of high levels of plasma ammonia resulting from high protein intake on conceptus survival and growth have not yet been fully recognized by swine producers. Compared with a 12%-CP diet, feeding a higher-protein diet reduced the number of live piglets born per litter and the number of piglets weaned per litter (Table 1). Likewise, a lower-protein diet (10% CP) is not optimal for pregnancy outcomes in gilts, due to the inadequate provision of AAs.

**Table 1:** Concentrations of amino acids (AAs) and metabolites in maternal plasma and reproductive performance of gestating gilts fed diets supplemented with 10–16% crude protein (CP)^1^

Variable	10% CP	12% CP	14% CP	16% CP	Pooled SEM
Concentrations of AAs and metabolites in maternal plasma					
Arginine, μmol/L	189^d^	204^c^	221^b^	238^a^	3.5
Cystine + cysteine, μmol/L	234^d^	268^c^	291^b^	314^a^	4.2
Glutamate, μmol/L	85	89	92	94	2.6
Glutamine, μmol/L	352^d^	378^c^	395^b^	417^a^	5.4
Glycine, μmol/L	608^d^	631^c^	662^b^	698^a^	7.3
Leucine, μmol/L	144^d^	162^c^	189^b^	208^a^	4.0
Lysine, μmol/L	112^d^	131^c^	150^b^	174^a^	2.8
Methionine, μmol/L	36^d^	43^c^	55^b^	62^a^	1.5
Ornithine, μmol/L	64^d^	78^c^	92^b^	116^a^	2.4
Proline, μmol/L	240^d^	276^c^	304^b^	345^a^	5.6
Serine, μmol/L	146^d^	163^c^	185^b^	207^a^	2.9
Tryptophan, μmol/L	41^d^	53^c^	64^b^	77^a^	1.1
Ammonia, μmol/L	56^d^	70^c^	81^b^	93^a^	1.7
Urea, mmol/L	1.67^d^	2.02^c^	2.46^b^	2.88^a^	0.061
Reproductive performance of gilts					
Total piglets born per litter, n	10.72	10.98	10.83	10.76	0.089
Total piglets born alive per litter, n	9.62^bc^	9.95^a^	9.74^b^	9.48^c^	0.067
Average birth weight of all piglets born, kg	1.33	1.36	1.35	1.34	0.012
Average birth weight of all piglets born alive, kg	1.34	1.37	1.36	1.35	0.011
Total litter weight at birth for all piglets born, kg	14.2^b^	14.8^a^	14.5^ab^	14.3^b^	0.14
Total litter weight at birth for all live piglets, kg	12.7^c^	13.5^a^	13.1^b^	12.6^c^	0.11
Piglets born dead per litter, n	1.10^b^	1.03^b^	1.09^b^	1.28^a^	0.048
Variations in birth weights among all piglets born,^2^%	18.3^a^	17.1^b^	18.1^a^	18.7^a^	0.30
Variation in birth weights among all piglets born alive,^2^%	16.1^a^	15.0^b^	16.4^a^	16.8^a^	0.28
Survival and growth of live-born piglets before weaning					
Milk intake of sow-reared piglets,^3^ mL/kg BW per day	179	184	186	180	7.4
Total piglets weaned per litter, n	8.46^c^	9.04^a^	8.76^b^	8.53^c^	0.053
Total litter weight at weaning (21 days of age), kg	45.2^c^	49.0^a^	47.3^b^	46.0^c^	0.36

^1^Data are means with pooled SEM, from the authors’ own work. There were 30 gilts per treatment group. During the entire gestation, each gilt (Yorkshire × Landrace dams and Duroc × Hampshire sire) was fed 2 kg/d of a corn- and soybean meal-based diet [130] in two equal meals at 0700 and 1800 h. The four gestation diets contained different CP content by varying the ratios of corn grain to soybean meal, and were made isocaloric (12.9 MJ/kg) with an appropriate addition of cornstarch. The body weight of gilts at breeding was 116 ± 0.9 kg, *n* = 120). Blood samples (~0.1 mL) were obtained from the ear vein of each gilt at d 110 of gestation at 2 h after feeding for analysis of metabolites in plasma [131]. Duration of gestation did not differ (*P* > 0.05) among the four groups of (114 ± 0.1 d, *n* = 120). During the entire lactation period, all sows had free access to the same corn- and soybean meal-based diet containing 18.2% CP [132]

^2^Coefficient of variation (SD/mean × 100%)

^3^On d 21 of lactation, milk consumption by piglets was determined by using the weigh-suckle-weigh technique [133]

^a-d^Within a row, means not sharing the same superscript letters differ (*P* < 0.05), as analyzed by one-way analysis of variance and the Student-Newman-Keuls multiple comparison [134]

### Negative impacts of maternal overnutrition during gestation on lactation and fetal growth

Increased feed intake by sows during all or part of gestation has a negative effect on feed intake during lactation [13]. In multiparous sows, increasing dietary intakes of both protein and energy by 43% during the first 50 days of gestation, relative to a standard gestational diet (10.7 MJ of DE/kg and 12.0% CP), decreases the birth weights of the 2 lightest and 2 heaviest piglets in litters [60]. Although overfeeding both energy and protein between d 25 and d 50 of gestation has no beneficial effect on muscle fiber number in the offspring, this nutritional treatment reduces the skeletal-muscle weight of newborn piglets due to a smaller fiber size [61]. However, the high intake of dietary protein during the whole pregnancy period decreases the number of muscle fibers at birth in pigs [54]. Furthermore, overfeeding gilts by 40% of the NRC requirements [62] during the entire gestation impairs fetal development and postnatal survival [13]. Thus, overfeeding during all or part of the gestational stage has a detrimental effect on pregnancy outcomes in swine and must be avoided in feeding practices.

## Impacts of IUGR on pig production

### Effects of IUGR on growth performance and feed efficiency in postnatal offspring

Lean-tissue growth results primarily from protein accretion in skeletal muscle, whereas fat deposition in pigs occurs mainly in WAT. Therefore, it is important to understand the developmental biology of these two tissues. Myocytes and adipocytes are derived from a common mesenchymal precursor [63]. Therefore, excess amounts of WAT are developed at the expense of skeletal muscle when embryonic myogenesis is impaired [64]. There are two developing muscle fibers in fetal pigs (Table 2): 1) primary fibers, formed by the rapid fusion of primary myoblasts between d 25 and d 50 of gestation, and 2) secondary fibers, formed on the surface of primary fibers between d 50 and d 90 of gestation [65]. The stages of their fetal and postnatal development are summarized in Table 2. The numbers of secondary muscle fibers, but not primary muscle fibers, are affected by the uterine environment [66, 67]. Likewise, IUGR influences the expression of proteome in fetal skeletal muscle [67]. Because the total number of muscle fibers is fixed at birth, their prenatal development is a major factor regulating the postnatal growth of offspring [61].

**Table 2:** Stages of the fetal and postnatal development of porcine skeletal muscle

Stage	Days of gestation	Major events
1	From conception to 25 d of gestation	Embryonic myogenesis from a common mesenchymal precursor
2	From 25 to 50 d of gestation	Formation of primary muscle fibers (rapid fusion of primary myoblasts)
3	From 50 to 90 d of gestation	Formation of secondary muscle fibers (formed on the surface of primary fibers)
4	From 90 to 95 d of gestation	Establishment of muscle fiber numbers
5	d 114 of gestation	Total numbers of muscle fibers are fixed at birth
6	After birth	Growth of skeletal muscle by increasing the size of its fibers (hypertrophy)^a^; and maturation of skeletal muscle

^a^Hypertrophy is defined as an increase in the size of the skeletal-muscle cell (also known as fiber), whereas hyperplasia refers to an increase in the number of cells or fibers

Adapted from Dwyer et al. [66], Handel and Stickland [65], Nissen et al. [61], and Oksbjerg et al. [42]

As noted previously [42, 67], the total number of skeletal muscle fibers at birth is lower in IUGR newborn pigs than in their littermates with a normal birth weight. This limits the extent of compensatory growth in these offspring [68]. Thus, there are differences in prenatal and postnatal growth rates between IUGR piglets and normal litter mates, which correlate with a lower ratio of secondary to primary muscle fibers and a smaller size of the fibers in IUGR pigs [65]. Reduced protein deposition in skeletal muscle and increased fat accretion in IUGR fetuses or offspring result from the abnormal metabolic regulation of intracellular protein turnover, adipogenesis, and mitochondrial biogenesis [5]. Consistent with this notion, the results of proteomic analysis have shown that newborn IUGR piglets have a greater abundance of proteasome (a complex of proteolytic enzymes for nonlysosomal protein degradation) in skeletal muscle and liver, but less eukaryotic translation initiation factor-3 (a key requirement for protein synthesis) in skeletal muscle, compared to piglets with a normal birth weight [41]. Consequently, when compared to piglets with a normal birth-weight, IUGR piglets exhibit 5–10% lower rates of daily weight gain and 5–10% lower feed efficiency (weight gain:feed intake) during the preweaning period [68–71].

The impact of birth weight on BW growth decreases as age increases (Table 3). This can be explained, in part, by increased fat accretion with age in both IUGR and normal-birth-weight pigs. The conversion of dietary fat to TAG in the body, which is associated with no water retention in tissues, confers a lower feed efficiency (weight gain/feed intake) than the conversion of dietary protein into protein in pig muscle. Low feed efficiency in IUGR piglets may result from not only less skeletal-muscle fibers but also suboptimal mitochondrial function in skeletal muscle [72], as well as the impaired development of the small intestine for nutrient digestion and absorption [73]. Thus, in assessing the effects of IUGR on feed efficiency for lean-tissue gains, the proportions of skeletal muscle and WAT, rather than changes in body weight alone, should be determined.

**Table 3:** Impacts of IUGR on growth and feed efficiency decrease with increasing age in pigs

Body weight	Total variance in ADG accounted for by BBW	Difference in ADG between IUGR pigs (1-kg BBW) and and large-birth-weight pigs (2-kg BBW)			Daily feed intake		Difference in gain: feed ratio between IUGR pigs (1-kg BBW) and large-birth-weight pigs (2-kg BBW)		
		Gilts	Barrows	Both sexes	IUGR pigs with 1-kg BBW	Large-birth weight pigs with 2-kg BBW	Gilts	Barrows	Both sexes
kg	%	g/d			kg/d		(kg/kg)		
46.7	12–13	83.4	81.4	82.4	1.62	1.63	0.051	0.050	0.050
64.6 kg	8–9	72.9	69.7	71.3	2.02	2.05	0.036	0.034	0.035
83.5 kg	4.7–5.3	39.7	53.0	46.4	2.30	2.34	0.017	0.023	0.020
102.5 kg	2.0–2.4	41.8	44.3	43.1	2.47	2.52	0.017	0.018	0.017

Adapted from Schinckel et al. [71]. This study involved 991 gilts and 977 barrows. BBW accounted for 14.4 and 13.0% of the variation in 158-d body weight in gilts and barrows, respectively. BBW accounted for 10.8 and 10.4% of the variation in 125-kg body weight in gilts and barrows, respectively. At 158-d body weight, gilts with 1-kg BBW had 10.6 kg less body weight than gilts with 2-kg BBW, whereas barrows with 1-kg BBW had 10.9 kg less body weight than barrows with 2-kg BBW. At the market weight (125-kg body weight), pigs with 1-kg BBW had 1% less lean tissue than pigs with 2-kg BBW. Gilts with 1-kg BBW require 13.3 more days to reach 125-kg body weight than gilts with 2-kg BBW, whereas barrows with 1-kg BBW require 12.6 more days to reach 125-kg body weight than barrows with 2-kg BBW. At the same body weight, daily feed intake did not differ between pigs with 1- and 2-kg BBW

*ADG* average daily gain; *BBW* birth body weight; *DFI* daily feed intake

Without any effective nutritional interventions, IUGR piglets will not be able to achieve catch-up growth at an absolute rate comparable to that of age-matched piglets with a normal birth weight either before or after weaning [69, 74]. For example, a difference of 0.31 kg in birth weight (1.04 vs. 1.35 kg) or of 0.8 kg in body weight at weaning (21 d of age) translates into an average difference of 4.4 days (159.3 vs. 154.9 d) from birth to the same market weight of 120 kg [74]. When muscle protein synthesis is reduced, dietary energy can be partitioned toward fat deposition within skeletal muscle and the WAT. In support of this view, the fat content of market-weight carcasses from runt pigs is increased, compared with their large littermates [70]. However, the carcass composition or the final eating quality of the pork may not differ substantially among pigs with a birth weight of 0.8 and 2.5 kg when they are fed adequately and slaughtered at the same 120-kg market weight [74].

### IUGR and reproduction performance of offspring

Smaller female pigs have a smaller uterus and less uterine secretion than larger female pigs [38, 75]. Insufficient uterine capacity in size and function limits placental attachment and growth within the uterus, thereby reducing nutrient and gas exchange between the mother and her fetuses [14]. In addition, when compared to female pigs with a normal birth weight, those with IUGR exhibit a delay or failure to express estrus, conceive or farrow, as well as reduced preweaning and postweaning growth performance in affected offspring [3]. Such adverse effects of IUGR can be carried for up to three generations [3]. There is also evidence that IUGR delays fetal follicular development of the ovaries, as well as the onset of puberty in postnatal life, in comparison to pigs with a normal birth weight [76]. Furthermore, stressing pregnant sows daily between weeks 12 and 16 of gestation (5 min of restraint daily) delays the first estrus of the female offspring, compared to the offspring of the control, non-stressed sows (172 vs. 158 d of age) [77]. Similarly, in gilts, the age at puberty was negatively correlated with birth weight ranging from 1.13 to 1.98 kg [78]. Results of published studies have also indicated that the timing of under-nutrition during gestation has important effects on the development of the fetal reproductive system (e.g., hypothalamus, pituitary, and gonads) [79].

There is indirect evidence that neonatal nutrition affects subsequent reproductive function in pigs [55]. For example, gilts which were raised in litters of six pigs (6 piglets/litter) prior to weaning had more corpora lutea (an indication of ovulation rate) and more embryos at d 25 of gestation than gilts which were raised in litters of 12 piglets (12 piglets/litter) [80]. Likewise, boars raised in litters of six or less piglets (≤6 piglets/litter) reached puberty sooner and produced more sperm per ejaculate, compared with boars raised in litters of nine or more piglets (≥9 piglets/litter) [81]. Furthermore, Estienne and Harper [82] reported that adult boars with a birth weight of < 1.36 kg had lower sperm concentrations and less total sperm per ejaculate than adult boars with a birth weight of > 1.86 kg, suggesting that birth weight is also a determinant of reproductive potential in males. Collectively, impaired fetal or preweaning growth may result in suboptimal reproductive performance in both female and male swine.

## Fetal and neonatal programming of growth, development and feed efficiency

### Epigenetics as a mechanism of fetal programming

**Fig. 3:**
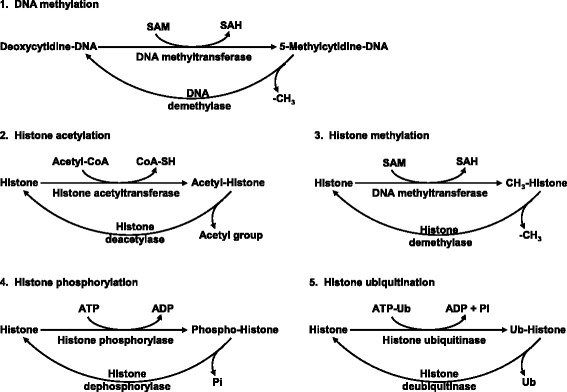
Biochemical reactions involving DNA methylation and histone modifications. These reactions are localized in specific compartments of the cell and are responsible for the epigenetic regulation of protein expression and function. Abbreviations: SAH, S-adenosylhomocysteine; SAM, S-adenosylmethionine; Ub, ubiquitin. Taken from Wang et al. [85]

The genetic code established by the DNA sequence is usually not altered after the formation of the diploid chromatin state at fertilization [83]. Changes in gene expression can be manifested by mitotically and/or meiotically heritable alterations in the DNA–protein complex without any change in the DNA sequence [84]. This phenomenon is known as epigenetics, with the Greek prefix “epi” meaning over or above. Molecular mechanisms responsible for the epigenetic regulation of protein expression and functions include: a) chromatin modifications; b) DNA methylation (occurring at the 5’-position of cytosine residues within CpG dinucleotides throughout the mammalian genome); c) histone modifications (acetylation, methylation, phosphorylation, ubiquitination, and sumoylation); and d) RNA-based mechanisms such as small noncoding RNAs or inhibitory RNAs (Fig. 3). The enzymes involved in these reactions include specific DNA and protein methyltransferases, DNA demethylases, GCN5-related N-acetyltransferase (a super family of acetyltransferase), histone acetyltransferase, histone deacetylase, histone demethylase, histone phosphorylase, histone dephosphorylase, histone ubiquitinase, and histone deubiquitinase [85].

Methylation is a key biochemical reaction affecting epigenetics, with the major donor of the methyl group being S-adenosylmethionine (a metabolite of methionine) [84]. Other AAs (histidine, glycine, and serine) and folate also participate in one-carbon metabolism to affect the provision of S-adenosylmethionine in cells [86]. The methyl group is donated from S-adenosylmethionine to the 5´-position of a cytosine nucleotide linked to a guanine nucleotide (CpG dinucleotide) by a phosphodiester bond. Regions with a high CpG dinucleotide content form CpG islands, which are strategically located in the regulatory regions of many genes, such as promoters and enhancers [87]. Consequently, changes in methylation status can either facilitate (hypomethylation for most genes) or inhibit (hypermethylation for most genes) the expression of genes. Epigenetic modifications are erased and re-established in a tissue-specific manner during embryonic development [88]. The haploid genomes of the sperm and oocyte possess different patterns of DNA methylation in a sex-specific manner [84]. After fertilization, the paternal genome is rapidly demethylated prior to the first cell division of the zygote. In contrast, the maternal genome is protected from this demethylation event, and, instead, is gradually demethylated during the development of the blastocyst. At the blastocyst stage, most methyl marks are removed, remaining present at the elements that regulate genomic imprinting and at retroviral elements [89]. After implantation, tissue-specific patterns of de novo DNA methylation occur. The processes of demethylation and remethylation during the development of germ cells are regulated by the phosphatidylinositol 3-kinase pathway [90].

Thus, through the methylation of genes, epigenetic information is heritable between cell generations [87]. Epigenetic transgenerational inheritance can be defined as germline-mediated inheritance of epigenetic information between generations that results in phenotypic variation [84]. In somatic cells, the methyltransferase, DNMT1 recognizes and methylates CpG dinucleotides present on the newly synthesized strand. As DNA synthesis occurs, the parental strand remains methylated, whereas the newly synthesized daughter strand does not. DNMT1 “reads” the parental strand and methylates CpG dinucleotides on the daughter strand that are complementary to methylated CpG dinucleotides on the parental DNA strand [88]. The critical role of DNA methylation in conceptus survival is made evident by the finding that all homozygous DNMT1 knockout mice die at the morula stage of embryonic development [91]. Epigenetic regulation of gene expression is the major mechanism responsible for the transgenerational effects of maternal nutrition on offspring [88].

### Genetic regulation of protein expression and cell signaling

Nutrition can regulate gene expression, micro-RNA biogenesis, and epigenetics in animal cells [6, 85]. For example, dietary glutamine reduces the intestinal expression of genes that promote oxidative stress and immune activation, while increasing the intestinal expression of genes that enhance cell growth and the removal of oxidants [92]. Consistent with its anti-oxidative and anti-fat deposition effects, dietary arginine inhibits the expression of key genes responsible for fatty acid synthesis, but stimulates the expression of key genes that are essential to fatty acid oxidation and glutathione synthesis in the WAT of rats [93, 94]. Dietary arginine also increases the expression of miRNA-15b/16 and miRNA-221/222 in the porcine umbilical vein to regulate angiogenesis and vascular remodeling [95]. Furthermore, glycine stimulates the intestinal expression of glycine transporter 1, while reducing the activation of the mitogen-activated protein kinase signaling pathway to confer anti-inflammatory effects [96].

Many AAs affect cell signaling via kinases [e.g., mammalian target of rapamycin (mTOR), AMP-activated protein kinase, cGMP-dependent kinase, cAMP-dependent kinase, and mitogen-activated protein kinase)], G protein-coupled receptors, and gaseous molecules (e.g., NO, CO and H_2_S) to regulate nutrient metabolism [97]. For example, dietary arginine enhances the abundance of the phosphorylated mTOR, eukaryotic initiation factor (eIF) 4E-binding protein-1 (4E-BP1), and ribosomal protein S6 kinase 1 (S6K1), as well as the formation of the active eIF4E-eIF4G complex, but reduces the abundance of the inactive 4E-BP1-eIF4E complex in skeletal muscle [98]. This leads to increased protein synthesis and whole-body growth [99]. Likewise, arginine or its metabolite putrescine activates mTOR to promote protein synthesis in placental cells and their proliferation [100]. In addition, dietary glutamine enhances intestinal integrity, cell survival, and villus height through the activation of mTOR cell signaling, while attenuating weaning-induced reduction in the abundances of occludin, claudin-1, zonula occluden (ZO)-2, and ZO-3 proteins [101]. Furthermore, dietary glutamate regulates the expression of glutamate receptors and taste receptor signaling in the pig’s gastrointestinal tract to maintain gut motility and function [102]. Likewise, AAs (e.g., arginine, cysteine and glycine) regulate the synthesis of NO, CO, and H_2_S, which participate in gaseous signaling in cells through cGMP and cAMP production to enhance blood flow, nutrient transport, anti-oxidative reactions, and immunity [103].

Because nutrients play a key role in gene expression, maternal or neonatal undernutrition can affect metabolic pathways and, therefore, cell growth, differentiation, and development [6]. This notion is supported by several lines of evidence. First, maternal consumption of a low-protein diet during pregnancy increases the expression and activity of the glucocorticoid receptor in the fetal liver, which may further enhance the capacity for gluconeogenesis in adults [10]. Of interest, maternal protein deficiency regulates mtDNA transcription in a sex-dependent manner [104], while increasing the hepatic expression of glucose-6-phosphatase in male piglets via histone methylation, acetylation and trimethylation, as well as micro-RNA biogenesis [105]. Second, maternal protein restriction alters the hepatic lipid content in male offspring [106], as well as a high incidence of fatty liver [107]. The latter may result, in part, from the decreased expression of peroxisomal proliferator-activated receptor-γ (PPARγ) through a micro-RNA-130b-dependent mechanism [108] and the increased expression of peroxisomal proliferator-activated receptor-α (PPARα) [109] in a tissue-specific manner. Third, maternal protein deficiency during gestation and lactation alters hepatic cholesterol metabolism in weanling piglets via the epigenetic regulation of expression of HMG-CoA reductase and cholesterol-7alpha-hydroxylase (CYP7α1) [110]. Furthermore, a deficiency of AAs affects the phosphorylation of eEF2 (eukaryotic elongation factor 2) kinase and eIF2α (eukaryotic translation initiation factor 2 alpha) through the general control nonderepressible 2 (GCN2) protein pathway, thereby inhibiting translation initiation for polypeptide formation [111, 112]. Thus, interactions betweern nutrients and gene expression is another basis for the adverse effects of malnutriton on animal growth, development, and health.

### Nutritional Interventions to Improve Embryonic, Fetal and Postnatal Survival and Growth

At first glance, augmenting dietary protein intake would seem to be a simple way to enhance swine fetal and neonatal growth under IUGR conditions. However, this approach has been reported to be detrimental to both fetal and neonatal survival. For example, when IUGR piglets were artificially fed a high-protein formula (50% more protein in comparison to sow’s milk) between 2 and 28 d of age, they exhibited poor growth and one-third of them died in association with hyperammonemia and elevated blood urea concentrations [113]. Thus, mechanism-based means should be developed to prevent and treat IUGR and its associated metabolic disorders.

### Embryonic, fetal and postnatal survival and growth

Because of ammonia toxicity, pregnant swine should not be fed a high-protein diet (e.g., ≥ 14% CP for gilts). There are reports that the prevention of maternal protein deficiency in mid-gestation sows (55–90 d) can increase the number of secondary fibers in their progeny [66] and, therefore, the rate of their growth and feed efficiency in the growing-finishing stage [42]. Thus, nutritional interventions critically depend on the gestation stage.

#### Arginine

As a functional AA, dietary supplementation with arginine is an effective means by which to improve pregnancy outcome in pigs [114]. First, dietary supplementation with 1.0% arginine-HCl between d 30 and 114 of gestation increases the number of live-born piglets by 2 and the litter birth weight by 24% [115]. The arginine-to-lysine ratio in the supplemental diet was 2.64, which did not affect the intestinal absorption of lysine or histidine. An arginine-to-lysine ratio of greater than 3:1 in the diet will result in antagonism among basic AAs and, therefore, should be avoided in dietary formulation [114]. Second, dietary supplementation with 1% arginine to gilts or sows between d 14 and d 28 of gestation increases the number of live-born piglets by approximately 1 at birth [116], whereas supplementation with 1% arginine to highly prolific gilts and sows between d 14 and d 28 of gestation enhances the number of fetuses on d 70 by 3 per litter [117]. Third, dietary supplementation with 0.4 or 0.8% arginine to gilts between d 14 and d 25 of gestation increases the number of live fetuses by 2 per litter [118]. The underlying mechanisms for the beneficial effects of arginine may involve: (a) the improved development or function of corpora lutea; (b) the maintenance of adequate production of progesterone, a major hormone for maintaining pregnancy; (c) enhanced placental NO and polyamine synthesis to promote placental angiogenesis and growth, and, therefore, the transfer of nutrients from the mother to her fetuses; and (d) improvements in the cellular redox state and anti-oxidative cell signaling [114, 119].

#### Glutamine

Glutamine is one of the most abundant AAs in fetal tissues and a major energy substrate for the fetus [15]. Because of a high rate of glutamine utilization by the rapidly growing fetus, the concentration of glutamine in the maternal plasma is reduced by 45% during late gestation in gilts, as compared to that during early gestation [120]. Inadequate provision of glutamine to the conceptus is likely a major factor contributing to IUGR in pigs. In support of this notion, we have found that supplementing 1% glutamine to the diet of gilts between d 90 and d 114 of gestation increases the birth weight and litter birth weight of live-born piglets, when compared with the isonitrogenous control [120]. Glutamine supplementation beneficially reduces the number of IUGR piglets, variations in birth weight, and preweaning mortality of live-born piglets by 39, 33, and 46%, respectively [120]. Thus, glutamine is an effective nutrient to enhance the productivity and performance of gestating pigs.

#### Arginine plus glutamine

Arginine also cooperates with glutamine to further improve the reproductive performance of pigs. The rationale for supplementation with arginine plus glutamine is that both AAs regulate protein synthesis by activating (a) the production of polyamines, which are essential for gene expression and mRNA translation, and (b) mTOR cells signaling. We have shown that adding 0.6% glutamine and 0.4% arginine to a corn- and soybean meal-based diet reduces (a) concentrations of ammonia (–29%) and urea (–27%) in maternal plasma (indicators of improved efficiency in the utilization of dietary AAs); (b) variation in birth weights among either all piglets born (–27%) or live-born piglets (–24%); and (c) the proportion of piglets with birth weights of 0.6 to 1.29 kg (–23% for all piglets born and –22% for live-born piglets) [5]. Furthermore, dietary supplementation with arginine plus glutamine increases (a) the number of live-born piglets by 1.4 per litter; (b) litter birth weight for either all piglets born (+10%) or live-born piglets (+15%), and (c) the proportion of piglets with birth weights of 1.3 to 1.49 kg (+37% for all piglets born and +30% for live-born piglets) (Wu et al. [5]).

The effects of arginine supplementation treatment in enhancing the porcine litter size and survival of porcine embryos/fetuses is expected to result in a tremendous economic return to swine producers. Specifically, an increase in the number of live-born pigs will markedly reduce production costs associated with reproduction and lactation in dams. Additionally, a reduction in the number of IUGR piglets will greatly improve the management of neonatal pigs and maximize preweaning survival and growth. Our findings provide a much needed basis for the dietary requirements of arginine and glutamine by gestating swine.

#### Folate

As noted previously, folate is essential for one-carbon metabolism, which is vital for the methylation of DNA and protein [86]. Thus, vitamins play an important role in embryonic survival and growth, as well as fetal and neonatal programming [118]. Of note, Randy Jirtle and his colleagues [121] discovered that maternal supplementation with folate can counteract bisphenol A-induced DNA hypomethylation and improve pregnancy outcomes in mice. Likewise, Waterland and Jirtle [122] demonstrated that adding a mixture of extra folate, betaine, choline, and vitamin B_12_ to the maternal diet of agouti mice affected the phenotype of their offspring via increasing CpG methylation at the A(vy) locus [122]. Consistent with the findings from the rodent studies, Liu et al. [123] have reported the differential expression of the proteins related to the metabolism of nutrients and oxidative stress in the livers of IUGR fetal pigs which have low concentrations of folate [124]. Interestingly, maternal supplementation with folic acid can alter the expression of proteins which are critical for immune and oxidative responses, as well as hepatic energy metabolism in newborn piglets [124]. The underling mechanisms likely involve the improvement of anti-oxidative signaling in hepatocytes and possibly other cell types.

## Postnatal survival and growth

Enterocytes are responsible for the endogenous synthesis of arginine in pigs [24] and, therefore, are critical for maximal growth of piglets and ammonia detoxification via the liver [1]. However, the development of the small intestine is compromised in IUGR pigs [73]. Thus, improving the integrity and function of the gut is an effective means to ameliorate neonatal death and growth restriction in IUGR offspring. For example, oral administration of glutamine (0.5 g/kg of BW twice daily; 1 g/kg of BW per day) between d 0 and d 21 of age enhances the growth of IUGR piglets by 16% while reducing their preweaning mortality by 48% [120]. Additionally, concentrations of ammonia in the plasma are 19% lower in the glutamine-supplemented IUGR piglets than in the control group [120], indicating that glutamine stimulates whole-body protein synthesis and inhibits whole-body AA oxidation. These findings have important implications for the nutritional management of compromised young pigs [125], improvement in the utilization of dietary protein for lean tissue growth [126], and the sustainability of global pork production [127]. While leucine supplementation can enhance intestinal development and growth, as well as whole-body growth in piglets with a normal birth weight [128], this method has a detrimental effect in IUGR piglets for yet unknown reasons [129]. Thus, the mechanisms responsible for the high rate of mortality in IUGR pigs should be further elucidated so that effective means will be developed to save their lives and enhance their productivity.

## Conclusion

In summary, a large body of evidence shows that maternal under- or over-nutrition and other environmental stresses immediately before breeding or during pregnancy (particularly early gestation) negatively influence the metabolism, growth and development of the porcine fetus, as well as numerous metabolic pathways, feed efficiency, and the disease susceptibility of the affected offspring. The underlying mechanisms involve the epigenetic regulation of gene expression, key transcription factors, protein abundance and activity, and multiple signaling pathways. Nutritional interventions involving dietary supplementation with functional AAs (e.g., arginine and glutamine) or possibly certain vitamins (e.g., folate) related to one-carbon metabolism and the provision of methyl groups can help overcome the adverse effects of maternal fetal and neonatal programming on the growth performance, feed efficiency, and well-being of IUGR offspring. Optimal AA nutrition has great promise to improve fetal growth, development, and survival not only through cell signaling but also via the epigenetic regulation of protein expression and functions.
